# Clinical outcomes of rotator cuff repair with subacromial bursa reimplantation: a retrospective cohort study

**DOI:** 10.1016/j.jseint.2023.05.010

**Published:** 2023-06-03

**Authors:** James M. Gregory, Cristian Ybarra, Zean Liao, Manickam Kumaravel, Saagar Patel, Ryan J. Warth

**Affiliations:** a Department of Orthopedic Surgery, University of Texas Health Science Center at Houston, McGovern Medical School, Houston, TX, USA; b REDCap Cloud, Encinitas, CA, USA

**Keywords:** Subacromial bursa, Rotator cuff repair, Biologic augmentation, Rotator cuff healing, Mesenchymal stem cell, Bursa reimplantation

## Abstract

**Background:**

The subacromial bursa has been found to be a rich, local, source of mesenchymal stem cells but is removed for visualization during rotator cuff repair. Reimplantation of this tissue may improve rotator cuff healing. The purpose of this study is to evaluate clinical outcomes of rotator cuff repair with and without subacromial bursa reimplantation.

**Methods:**

Patients aged 37-77 with a full-thickness or near full-thickness supraspinatus tears underwent arthroscopic transosseous-equivalent double row rotator cuff repair. In patients prior to July 2019, the subacromial bursa was resected for visualization, and discarded. In patients after July 2019, the subacromial bursa was collected using a filtration device connected to an arthroscopic shaver and reapplied to the bursal surface of the tendon at the completion of the rotator cuff repair. Rotator cuff integrity was evaluated via magnetic resonance imaging on bursa patients at 6 months postoperatively. Minimum 18-month clinical outcomes (Single Assessment Numeric Evaluation, American Shoulder and Elbow Surgeons, patient satisfaction) were compared between bursa and nonbursa cohorts.

**Results:**

A total of 136 patients were included in the study (control n = 110, bursa n = 26). Preoperative demographics and tear characteristics were not different between groups. Average follow-up was significantly longer in the control group (control: 3.2 ± 0.7 years; bursa: 1.8 ± 0.3 years; *P* < .001). The control group showed a significantly higher Single Assessment Numeric Evaluation score (control: 87.9 ± 15.8, bursa: 83.6 ± 15.1, *P* = .037) that did not meet minimum clinically important difference. The American Shoulder and Elbow Surgeons and patient satisfaction scores were similar between the groups. Symptomatic retears were not significantly different between groups (control: 9.1%, bursa 7.7%, *P* = .86). Seven patients in the control group underwent reoperation (6.4%), compared to 0 patients in the bursa group (0%, *P* = .2). Six-month postoperative magnetic resonance images obtained on bursa patients demonstrated 85% rotator cuff continuity (n = 17/20) as defined via Sugaya classification.

**Conclusion:**

Augmentation of rotator cuff repair with bursal tissue does not appear to have negative effects, and given the accessibility and ease of harvest of this tissue, further research should be performed to evaluate its potential for improved tendon healing or clinical outcomes.

Rotator cuff injuries continue to represent one of the most frequently encountered soft-tissue pathologies in orthopedic surgery—up to 22% adult population over the age of 65 and up to 50% over the age of 80 are likely to have a chronic degenerative rotator cuff tear regardless of their symptomatology.^1^^,^^3^^,^^23^ In contrast to acute rotator cuff injuries (which have favorable prognoses due to good tissue quality and a favorable biologic healing environment), the postoperative course after repair of chronic degenerative cuff tears is much less predictable. Factors such as decreased tendon mobility, tendon retraction, tendon loss, fatty degeneration, and chronic insertional changes combine to create an insufficient mechanical and biologic environment to support healing. Although surgical repair of chronic tears has been shown to be successful, the rate of rotator cuff healing is variable and can range from 6% to 90% depending on patient and rotator cuff tear characteristics—the prognosis becomes even less favorable for those who require revision repair.^1^^,^^3^^,^^8^^,^^18^^,^^23^^,^^24^

Previous studies have demonstrated that traditional rotator cuff repairs result in disorganized fibrous scar tissue that weakly integrates with the tendon footprint.^4^^,^^18^,^19^ This disorganized scar tissue neither resembles the mechanical strength nor specialized histologic architecture of the native tendon insertion. Recent published literature suggests that strategic use of growth factors, cytokines, progenitor cells, and others may improve tissue quality at rotator cuff enthesis after surgical repair.^5^, ^6^, ^7^ Our group and others have demonstrated that the subacromial bursa, tissue that is normally discarded during arthroscopic shoulder surgery, contains an abundance of mesenchymal stem cells (MSCs)^11^^,^^22^ and that this tissue can be reimplanted into the site of rotator cuff repair in a single step.^13^^,^^16^ Specifically, the tight fibrovascular network, a high growth factor content, and the large progenitor potential of bursa-derived cells could complement the deficits that a nearby rotator cuff injury might experience due to the fact of its low endogenous regeneration potential.^10^

As a result, use of this tissue to augment rotator cuff repair appears^5^ promising. However, the subacromial bursa has previously been implicated as a pain generator within the shoulder and has been thought to be proinflammatory in certain conditions.^20^ It is possible that reimplantation of subacromial bursa tissue may cause an inflammatory response, resulting in increased pain, or potentially compromising rotator cuff healing.^17^ To our knowledge, although the technique of subacromial bursa reimplantation has been described, clinical results have not been reported as of this writing. Prior to the necessary high-quality randomized trials to determine whether bursa tissue is truly efficacious to improve rotator cuff healing, safety data and short-term clinical results must first be reported to justify larger clinical investigations.

The purpose of this study was to compare the clinical results of patients undergoing arthroscopic rotator cuff repair with concomitant reimplantation of minced subacromial bursa tissue to a control group of patients who did not receive bursal reimplantation. We hypothesized that reimplantation of subacromial bursa tissue at the time of rotator cuff repair would not have negative effects on healing or clinical outcomes as compared to a retrospective cohort that underwent repair without bursa reimplantation.

## Methods

### Patient selection

**Figure 1:**
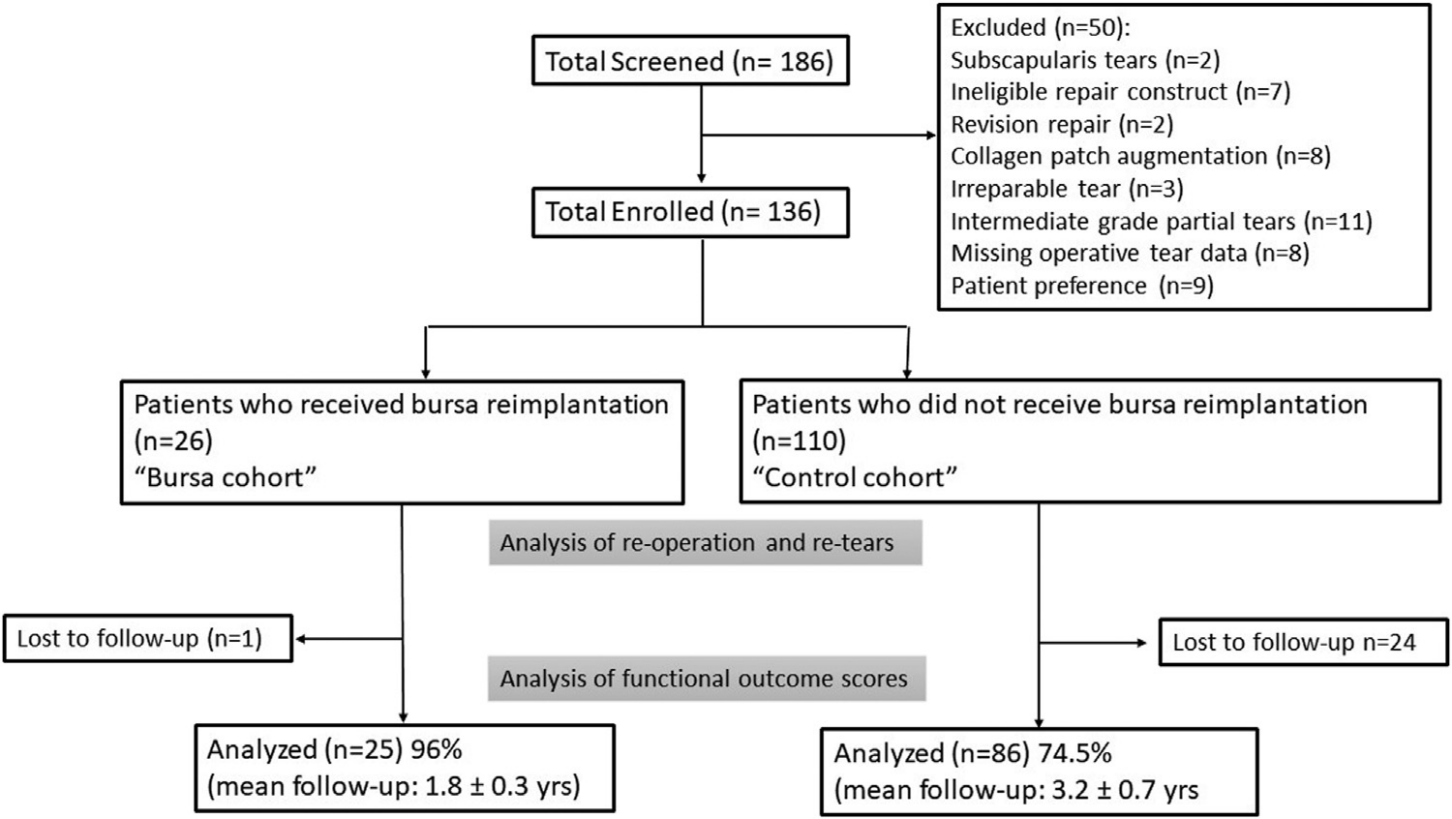
Flow diagram of study design and subject participation, including excluded patients and those lost to follow-up.

Local institutional review board approval was obtained prior to initiation of this retrospective study. All patients who underwent surgical repair of a full-thickness or near full-thickness rotator cuff tear by the senior surgeon (J.M.G.) were included. Near-full thickness tears were defined as tears in which only a very thin portion of rotator cuff tissue remained attached to the tuberosity, and in situ repair would not be possible. To minimize bias between the cohorts, consecutive patients were enrolled, and all patients received a transosseous-equivalent rotator cuff repair involving the supraspinatus. From January 4, 2017 to July 31, 2019, a subacromial bursectomy was performed to allow for visualization of the rotator cuff, and the bursa tissue was discarded. From August 1, 2019 until December 18, 2019, the bursa tissue was collected during the bursectomy and then reimplanted at the completion of the rotator cuff repair. Exclusion criteria included patients who did not receive a transosseous-equivalent repair, partial repairs, revision repairs, irreparable tears, isolated subscapularis repairs, those missing operative tear data, those who received other forms of biologic augmentation (ie, patches, platelet-rich plasma), and patients who refused to participate in the study (Fig. 1). Patients with systemic inflammatory conditions such as rheumatoid arthritis were not indicated for bursa reimplantation. No patients had to be excluded for this reason. Indications for rotator cuff repair construct, and biologic augmentation other than bursa reimplantation remained unchanged throughout the study period.

### Surgical procedure

The surgical technique for this procedure has previously been published.^16^ Surgery was performed in the beach chair position. Standard posterior and anterior portals were established, followed by diagnostic arthroscopy and assessment of the rotator cuff tear. An accessory lateral portal was established in preparation for tissue harvest. The subacromial bursa was minced and suctioned using a 4.5-mm arthroscopic shaver and collected for later reimplantation (GraftNet Autologous Tissue Collector; Arthrex Inc., Naples, FL, USA). The collected bursa was then loaded into a 3-cc syringe in preparation for reimplantation. In the retrospective cohort of patients that did not receive bursa reimplantation, the bursa was débrided and discarded without collection.

The rotator cuff repair was performed in an identical fashion in both cohorts. The rotator cuff footprint was prepared through decortication of the greater tuberosity insertion with an arthroscopic shaver and/or burr, followed by marrow stimulation with an awl. In accordance with the dimensions of the tear, 1-3 bioabsorbable suture anchors were inserted medially, and sutures were passed through the cuff tendon in a horizontal mattress configuration. The free limbs of each suture were then passed and secured to corresponding lateral anchors, establishing a double-row transosseous equivalent repair construct.

In the patients who received bursa reimplantation, arthroscopic fluid was evacuated from the shoulder. The 3-cc syringe preloaded with the minced subacromial bursa tissue is then placed into the lateral portal. The subacromial bursa tissue was reimplanted on the surface of the double-row repair construct. The fluid was not restarted after bursa reimplantation, and the arthroscopic portion of the case was completed at this point. Typical time required for preparation and reimplantation of bursal tissue was less than 5 minutes but was not directly measured as part of the study.

### Postoperative management

Patients were managed with a rotator cuff repair protocol in which sling use is required for 6 weeks, during which time physical therapy focuses on passive range of motion. Active range of motion was performed from 6-12 weeks, with gentle shoulder strengthening beginning at approximately 10 weeks.

### Data collection

All clinical data were retrieved retrospectively from medical records. Demographic information included age, gender, hand dominance, and operative side. Preoperative magnetic resonance imaging (MRI) was assessed for fatty infiltration using the Goutallier classification^4^ and anteroposterior tear width. Postoperative MRIs were obtained on patients who received bursa reimplantation at the 6-month mark. The images were assessed for repair integrity using Sugaya classification,^21^ where Sugaya grades 4 and 5 were considered to represent MRI-confirmed repair discontinuity. The degree of fatty infiltration was evaluated on dedicated sagittal oblique T1 weighted images. The confirmation of the repair integrity was evaluated using coronal T2 weighted proton density and also using heavily T2 weighed sagittal oblique images, so as to overcome magic angle artifact. All images were interpreted and classified by a Board-Certified musculoskeletal radiologist. Clinical outcomes including Single Assessment Numeric Evaluation (SANE), American Shoulder and Elbow Surgeons, and patient satisfaction scores were collected at minimum 18-month follow-up. Patient satisfaction was determined using an ordinal scale from 1 to 10 as a response to the question “How satisfied are you overall with the outcome of your surgery?”. 1^15^ Only those scores resulting from fully completed questionnaires were utilized. Patients in the control group who had pain and clinical findings concerning for retear received postoperative MRIs as clinically indicated. Symptomatic retears were defined as pain after rotator cuff repair with an imaging workup which revealed a rotator cuff tear. Retears and reoperations were collected and analyzed.

### Statistical analysis

Statistical analyses were performed using RStudio and SPSS (IBM Corporation; Armonk, NY, USA). Descriptive statistics are reported as averages with standard deviations and ranges for continuous variables. Chi-squared tests were used to compare categorical variables and Mann-Whitney *U* tests were used for continuous variables. Spearman's ρ and Pearson R correlation coefficients were calculated to identify potential correlations among categorial and continuous variables, respectively. Two-tailed *P* values less than .05 were considered statistically significant.

## Results

A total of 186 patients underwent arthroscopic rotator cuff repair during the study period and were screened for inclusion into the study. Fifty patients were excluded from the study, leaving a total of 136 study patients. One-hundred ten patients did not receive bursal reimplantation (control), and 26 received bursa reimplantation at the completion of the repair (bursa). Preoperative MRIs were available in all bursa patients, and 85/110 (77%) of control patients. The average tear width was 2.0 ± 1.1 cm in the control group and 1.8 ± 0.9 cm in the bursa group (*P* = .41). Preoperative patient demographics and tear characteristics are shown in Table I, and were not significantly different between the 2 cohorts.

**Table I:** Preoperative patient demographics and tear characteristics.

	Control (n = 110)	Bursa (n = 26)	*P* value
Age	59.8 ± 10.3	59.9 ± 9.3	.83
Side			.23
Left	32	11	
Right	78	14	
Dominant hand			1
Left	7	2	
Right	75	23	
Unknown	28	1	
	Control (n = 85)	Bursa (n = 26)	
Tear width (cm)	2.0 ± 1.1	1.8 ± 0.9	.41
Goutallier			.09
0	25	11	
1	23	11	
2	15	2	
3	8	2	
4	14	0	

Table II summarizes the clinical results of both cohorts at minimum 18-month follow-up. In the cohort group, 86/110 patients (74.5%) had complete collection of functional outcome scores, compared to 25/26 patients (96%) in the bursa group. Average follow-up was significantly longer in the control group (control: 3.2 ± 0.7 years; bursa: 1.8 ± 0.3 years; *P* < .001). The control group showed a significantly higher SANE score (control: 87.9 ± 15.8, bursa: 83.6 ± 15.1, *P* = .037). American Shoulder and Elbow Surgeons and patient satisfaction scores were similar between the groups. Retears and reoperations were evaluated among the entire cohort. Symptomatic retears were not significantly different between groups (control: 9.1%, bursa 7.7%, *P* = .86). Seven patients in the control group underwent reoperation (6.4%), compared to 0 patients in the bursa group (0%, *P* = .2). Reasons for reoperation in the control group were stiffness (n = 3), symptomatic retear (n = 3), and infection (n = 1). Not all patients with symptomatic retear opted for reoperation.

**Table II:** Clinical outcomes comparison.

	Control (n = 110)	Bursa (n = 26)	*P* value
Symptomatic retear	10 (9.1%)	2 (7.7%)	.86
Reoperations			.2
Yes	7 (6.4%)	0 (0%)	
No	103	26	
	Control (n = 86)	Bursa (n = 25)	

Follow-up (yrs)	3.2 ± 0.7	1.8 ± 0.3	<.001∗
Satisfaction	9.2 ± 1.8	9.2 ± 1.26	.31
SANE	87.9 ± 15.8	83.6 ± 15.1	.037∗
ASES	89.9 ± 16.6	84.8 ± 21.0	.07

*SANE*, Single Assessment Numeric Evaluation; *ASES*, American Shoulder and Elbow Surgeons.

Six-month postoperative MRI was available for 20/26 bursa patients (76.9%) and is listed in Table III. MRI-confirmed repair discontinuity was found in 3/20 patients at 6-month follow-up (15.0%), defined as Sugaya grades 4 and 5. Two of these patients sustained traumatic events. Both of these patients had symptomatic retears but opted for nonoperative treatment.

**Table III:** Postoperative MRI appearance of bursa cohort.

Sugaya class	Patients (n = 20)
Class 1	1
Class 2	8
Class 3	8
Class 4	2
Class 5	1

*MRI*, magnetic resonance imaging.

## Discussion

The results of this retrospective study suggest that augmentation of rotator cuff repair with bursal tissue does not appear to have clinically relevant negative effects at minimum 18-month follow-up, and clinical outcomes were similar to a retrospective cohort who received identical rotator cuff repair technique and indications. The SANE score was significantly higher in the control group, but this difference of 4.3 points is unlikely to be clinically meaningful, as it is much lower than the minimum clinical important difference of 13.0 established by Kim et al for the rotator cuff repair population.^9^ Additionally, there was no significant difference in symptomatic retear and reoperation rates in the bursa cohort. It is possible that our study is underpowered to detect differences in clinical outcomes, or that bursal reimplantation does not affect healing rates. However, the goal of this study was to evaluate the clinical outcomes of using bursal tissue during rotator cuff repair given the concern that this tissue may cause a proinflammatory response affecting healing. This study illustrates that use of bursal tissue does not have a clinically relevant negative effect and does not compromise outcomes at short-term follow-up. Although selection bias is a concern with any form of retrospective cohort study, our study is generalizable, and attempts were made to minimize this bias. The patients who received bursa reimplantation were selected consecutively, and the technique and indications for rotator cuff repair remained unchanged between these patients and the retrospective control cohort.

Clinically, it can be observed that acute, traumatic rotator cuff tears are often accompanied by proliferative, vascular, subacromial bursal tissue. In chronic degenerative tears, this bursal tissue is commonly much less proliferative and may be much less present. One can hypothesize, as a result that the proliferation of bursal tissue may represent a healing response to underlying rotator cuff injury. Basic science observations have begun to support this concept, and have led to increasing interest in the use of subacromial bursa tissue as a biologic augment to rotator cuff healing. The subacromial bursa has been found to be a rich source of MSCs that are capable of differentiating in vitro into a variety of mesenchymal phenotypes, including muscle, tendon, and bone.^11^^,^^13^^,^^14^^,^^22^ The inherent pluripotency of subacromial bursa cells persists regardless of patient demographics or rotator cuff characteristics,^14^ while also appearing to exhibit greater differentiation ability, and better engraftment into tendon repair models than concentrated bone marrow aspirate.^2^

There is significant interest in accelerating research in the area of cellular therapies for a variety of musculoskeletal conditions. Many of these promising cellular therapies, however, are often costly and require multiple surgical procedures to complete. The use of subacromial bursa for augmentation of rotator cuff repair has significant advantages in that the tissue source is readily accessible through the same surgical incision, does not require laboratory expansion prior to reimplantation, and can be reimplanted during the same surgical procedure. This approach could potentially allow patients to receive cellular treatments without creating significant cost barriers or additional surgical risks.

While there are obvious advantages to this approach, it remains unknown how the subacromial bursa tissue behaves in vivo when used in conjunction with rotator cuff repair. As the bursa has been implicated as a proinflammatory pain generator in the shoulder, it is possible that surgical reimplantation of this tissue may cause increased inflammation resulting in pain, stiffness, or impaired tendon healing. As a result, understanding the safety profile of subacromial bursa reimplantation during rotator cuff repair is essential prior to undertaking larger clinical studies. Within the bursal cohort in this study, we report no reoperations, a high patient satisfaction rate, a low rate of symptomatic retears (7.7%, 2/26 patients), and a low rate of MRI-confirmed repair discontinuity 6 months after surgery (15%, 3/20 patients). This suggests that reimplantation of bursal tissue during rotator cuff repair does not have clinically relevant negative effects. Not unexpectedly, patient age was significantly positively correlated with Goutallier grade. These results are consistent with prior clinical studies evaluating the clinical outcomes after arthroscopic rotator cuff repair.^12^^,^^23^

A limitation of this study is that we only have postoperative MRIs on the patients who received bursal reimplantation. Our standard practice is that we do not routinely image patients after rotator cuff repair in the absence of clinical indication. No external funding was involved in this study—MRIs in our system are paid for via insurance carriers and are not covered as part of routine postoperative care. As a result, the control group did not routinely receive postoperative MRIs. However, 6-month MRIs were ordered on the bursa cohort as these represented the first patients in which this bursal reimplantation technique was performed. As we did not identify a concerning early failure rate, we have since returned to the practice of ordering postoperative imaging only when clinically indicated.

These results are short-term and may not reflect long-term surgical outcomes. Postoperative MRI evaluation of the rotator cuff at 6 months does not represent the completion of the healing process, and it is possible that the Sugaya grade may continue to change through the 1-year time point. However, it is well-reported that the majority of rotator cuff healing failures and complications occur by 6 months postoperatively, so we felt that this was an appropriate time point to evaluate these preliminary outcomes given the focus of our study.

Much remains unknown about the behavior of subacromial bursa tissue in vivo, and the optimal preparation and delivery of subacromial bursa tissue during rotator cuff repair remains unknown at this time. Our technique involves capturing the subacromial bursa tissue after it has been mechanically minced using a standard arthroscopic shaver, and reimplantating it onto the bursal surface of the rotator cuff. It has been shown that mechanical, nonenzymatic bursal tissue processing such as this improves nucleated cell count and preserves MSC viability compared to the use of whole bursal tissue.^13^ We chose to reimplant the minced tissue on the bursal surface of the rotator cuff to better mimic the native position and function of the subacromial bursa, and to minimize the risk of an unknown biologic intervention. However, it is possible that reimplantation is more effective at the tendon/bone interface. Further studies will be needed to define the optimal positioning of bursal tissue at the repair site, as well as survival and/or behavior of the bursal tissue after reimplantation.

## Conclusion

Augmentation of rotator cuff repair with bursal tissue does not appear to have a clinically relevant negative effect, and given the accessibility and ease of harvest of this tissue, further research should be performed to evaluate its potential for improved tendon healing or clinical outcomes.

## Disclaimers

Funding: No funding was disclosed by the authors.

Conflicts of interest: James M. Gregory reports that he has received consultant agreement/research support from Stryker and Arthrex; and stock/stock options from Sparta Biomedical for work related to the subject of this article. All the other authors, their immediate families, and any research foundations with which they are affiliated have not received any financial payments or other benefits from any commercial entity related to the subject of this article.
